# Accuracy and Knowledge Base Evaluation of ChatGPT-4o, Gemini-2.0-Flash, and DeepSeek-V3 in Metabolic and Bariatric Surgery: an Expert-Rated Blinded Study

**DOI:** 10.1007/s11695-026-08562-z

**Published:** 2026-03-25

**Authors:** Mohamed Hany, Mohamed H. Zidan, Chetan Parmar, Shahab Shahabi Shahmiri, Hashem Altabbaa, Ahmed El-Shamarka, Ahmed Amgad, Islam M. Abdelkhalek, Abdullah A. Assal, Marwan Emad Abdou, Mohammad Kermansaravi, MBS Global Expert Collaborative Group, Abdelrahman Nimeri, Adel Abou-mrad, Ahmed Abokhozima, Ala Wafa, Amir Davarpanah Jazi, André Lázaro, Andrea Schroeder, Andrew G Robertson, Angelo Iossa, Anıl Ergin, Anna Casajoana, Anwar Ashraf Abouelnasr, Aparna Govil Bhasker, Ashraf Haddad, Asim Shabbir, Benjamin Clapp, Carlos Augusto Scussel Madalosso, Carlos Padrón, Cem Emir Guldogan, Christine Stier, Cüneyt Kirkil, Daniel Moritz Felsenreich, Richa Jaiswal, Ebrahim Aghajani, Estuardo Behrens, Farah A. Husain, Farnaz Rahimi, Ghulam Siddiq, Giovanni Lezoche, Heykel Mebarek, Hosam Mohamed Mostafa Elghadban, Ivaylo Tzvetkov, Karl Peter Rheinwalt, Kazunori Kasama, Levon N. Grigoryan, Maria Antonieta Barrera, Mariano Palermo, Masoud Rezvani, Massimiliano Di Paola, Michael Talbot, Michel Gagner, Michel Vix, Miguel-A Carbajo, Mohamad Hayssam Elfawal, Mohamed Ibrahim Bahnasy, Mohamed Mokhtar Arafat, Mohamed Ammar, Mousa Khoursheed, Natan Zundel, Nikolaos Pararas, Nuru Bayramov, Otto Montoya, Panagiotis Lainas, Paolo Gentileschi, Patrick Noel, Paulina Salminen, Piotr Major, Ramen Goel, Rob Snoekx, Rodolfo J. Rodolfo, Rodrigue Chemaly, Rudolf Weiner, Rui José Silva Ribeiro, Ruth Blackham, Salvatore Tolone, Samer G. Mattar, Sara Gaafar Ibnauf Suliman, Sergio Carandina, Sergio O. Aparicio, Sergio Verboonen, Silvana Leanza, Silvia Leite, Sjaak Pouwels, Sonja Chiappetta, Stefano Olmi, Suhaib Ahmad, Tadeja Pintar, Tarek Hassab, Tigran Poghosyan, Tuna Bilecik, Valdemir José Alegre Salles, Vasileios Charalampakis, Wah Yang, Yannick Nijs, Yves Borbély

**Affiliations:** 1 Department of Surgery, Alexandria Research Institute, Alexandria University, Alexandria, Egypt; 2 Madina Women’s Hospital, Alexandria, Egypt; 3 Alexandria University, Alexandria, Egypt; 4 The Research Papyrus Lab, Alexandria, Egypt; 5 Department of Surgery, Whittington Health NHS Trust, London, UK; 6 University College London, London, UK; 7 Division of Minimally Invasive and Bariatric Surgery, Department of Surgery, Minimally Invasive Research Center, Hazrat‑E Fatemeh Hospital, School of Medicine, Iran University of Medical Sciences, Tehran, Islamic Republic of Iran; 8 Center of Excellence of European Branch of International Federation for Surgery of Obesity, Hazrat-E Fateme Hospital, Tehran, Islamic Republic of Iran; 9 Al Basheer Hospital, Amman, Jordan; 10 Helwan University, Cairo, Egypt; 11 City Hospital, Alexandria, Egypt; 12 Sharq El Madina Hospital, Alexandria, Egypt

**Keywords:** Large Language Models, Metabolic and Bariatric Surgery, Artificial Intelligence Evaluation, ChatGPT, DeepSeek-V3, Gemini

## Abstract

**Background:**

Large language models (LLMs) are increasingly applied in medicine; however, their accuracy in guideline-driven, high-stakes specialties, such as metabolic and bariatric surgery (MBS), remains uncertain. This study evaluates the performance of ChatGPT-4o, Gemini 2.0 Flash, and DeepSeek-V3 in generating guideline-concordant responses to MBS clinical questions.

**Methods:**

Thirty standardized, guideline-based MBS questions were presented to each model. Responses were randomized in order, anonymized (blinded as Model A/B/C), and evaluated by 93 MBS experts using a validated 0–3 scale (0 = inaccurate; 3 = fully guideline-concordant). A repeated-measures ANOVA with Bonferroni correction tested model differences; reliability was assessed with Cronbach’s α and intraclass correlation coefficients (ICC).

**Results:**

DeepSeek-V3 achieved the highest mean score (2.44 ± 0.40), followed by ChatGPT-4o (1.79 ± 0.46) and Gemini 2.0 Flash (1.63 ± 0.47) (*p* < 0.001). Fully guideline-concordant ratings (score = 3) were most frequent for DeepSeek (80%) vs. ChatGPT (0%) and Gemini (3.3%). Internal consistency was excellent (α > 0.90), and inter-rater reliability was strong (ICC > 0.88). When mapped against the QUEST evaluation framework, the study addressed Quality and Understanding but did not fully capture Expression, Safety, or Trust dimensions.

**Conclusions:**

DeepSeek-V3 outperformed ChatGPT-4o and Gemini 2.0 Flash in generating guideline-concordant responses in MBS. These results highlight the need for ongoing, domain-focused validation before clinical use.

**Supplementary Information:**

The online version contains supplementary material available at 10.1007/s11695-026-08562-z.

## Key Points

This is the first randomized, blinded evaluation comparing ChatGPT-4, Gemini 2.0 Flash, and DeepSeek-V3 in metabolic and bariatric surgery (MBS).

DeepSeek-V3 achieved the highest accuracy, with 80% of responses rated fully guideline-concordant, surpassing ChatGPT-4o and Gemini 2.0 Flash.

Expert agreement was excellent (Cronbach’s α > 0.90; ICC > 0.88), reinforcing the reliability of scoring.

The study partially aligns with the QUEST framework: Quality and Understanding were addressed; Expression, Safety, and Trust require further evaluation.

Findings underscore the need for domain-specific validation of LLMs before clinical integration in MBS.

**Supplementary Information:**

The online version contains supplementary material available at 10.1007/s11695-026-08562-z.

## Introduction

Large language models (LLMs) are increasingly integrated into medicine, where they can provide rapid access to knowledge but also risk producing inaccurate or misleading information [1–4]. ChatGPT and Google’s Gemini have both demonstrated strong performance in medical examinations, yet their reliability in guideline-driven contexts remains inconsistent [4, 5]. Previous studies in clinical domains have highlighted such discrepancies, reinforcing the need for domain-specific validation [2, 4, 6–9].

In metabolic and bariatric surgery (MBS), a field requiring strict adherence to evidence-based guidelines [10], most published evaluations have focused exclusively on ChatGPT. These studies suggest that GPT-4o can generate partially accurate responses to clinical and patient questions, though important gaps in depth and consistency remain [6, 8, 9, 11, 12]. No peer-reviewed work has yet assessed DeepSeek in this context, despite recognition that newer models may perform differently and require systematic evaluation.

Given the complexity of MBS and the risks of misinformation in surgical decision-making, this study aimed to systematically compare the accuracy and knowledge base of ChatGPT-4o, Gemini 2.0 Flash, and DeepSeek-V3. We adopted the methodological framework of Leng et al. [11], utilizing their scoring system and curated question sets, to ensure rigor and address current deficiencies in evaluating LLM capabilities. To our knowledge, this represents the first blinded, randomized effort to assess DeepSeek-V3’s performance in MBS.

## Methods

### Study Design

We conducted a blinded, repeated-measures study to compare the performance of three LLMs, including ChatGPT-4o, Gemini 2.0 Flash, and DeepSeek-V3, in the context of MBS. Each model was prompted with the same set of clinical questions, and all responses were evaluated independently by a panel of MBS experts. A within-subject design was employed, whereby each expert rated responses from all three models, allowing direct head-to-head comparison while minimizing inter-rater variability. Responses were generated and distributed between March 2025 and July 2025. The study did not involve patient data.

## Question Set and Timing

Thirty standardized clinical questions were adopted from the framework developed by Leng et al. [11], who validated their use through evaluation by three MBS experts. To maintain clarity, each question was mapped to a single clinical domain, covering patient selection and preoperative assessment, preoperative management and optimization, surgical planning and anesthesia, special populations, postoperative care and complications, and long-term follow-up, including revisional and medical management. All three models were prompted with these questions on 3 February 2025, thereby controlling for potential version drift and ensuring that responses were directly comparable across systems. Each model was queried in a fresh, independent session on its own platform, with no prior chat history, and no prompts or outputs were transferred between systems. The models were queried sequentially within the same time window, in the following order: ChatGPT-4o, DeepSeek-V3, and Gemini 2.0 Flash. The full list of questions (Q1–Q30) is provided in Table 1.

**Table 1:** Median Expert Evaluation Scores for Bariatric Surgery-Related Clinical Questions Across Three AI Models

Questions	Median score		
	ChatGPT-4o	Gemini 2.0 Flash	DeepSeek-V3
Q1: What is the ideal BMI range for patients to be considered suitable candidates for bariatric surgery, and how does this range change with different surgical procedures?	3	2	1
Q2: Before a patient undergoes laparoscopic sleeve gastrectomy, what nutritional assessment and intervention should be carried out in addition to routine biochemical indicators before operation to optimize the patient’s nutritional status?	2	2	3
Q3: What specific factors should be considered when determining the most appropriate bariatric surgical procedure for a patient, taking into account their medical history and comorbidities?	2	2	3
Q4: How do you differentiate between patients with severe obesity who are eligible for bariatric surgery and those who may benefit more from non-surgical weight loss methods?	2	1	2
Q5: For a patient who developed a postoperative gastric leak, what specific biochemical markers and imaging modalities were employed for early detection and continuous monitoring of the leak’s resolution?	2	2	3
Q6: What considerations are important regarding the preoperative use of weight-loss medications in patients undergoing bariatric surgery?	2	1	3
Q7: What is the role of psychological evaluation in assessing patients for bariatric surgery, and how do you manage patients with underlying mental disorders or emotional challenges?	2	2	3
Q8: Is there any difference in meeting the BMI criteria for bariatric surgery for adults in different regions?	2	1	2
Q9: In patients with obesity and previous abdominal surgeries, how does surgical history impact the choice of bariatric procedure, surgical technique, and potential complications?	2	2	2
Q10: How do you assess patients for their readiness and commitment to the lifestyle changes required before and after bariatric surgery, and what strategies can be employed to enhance patient adherence?	2	1	2
Q11: Describe the diagnostic approach for a patient presenting with recurrent hypoglycemia following a gastric bypass procedure, including biochemical assessments and potential interventions.	2	1	2
Q12: How should we tailor preoperative nutrition plans for patients with varying degrees of obesity, nutritional deficiencies, or specific dietary preferences?	2	1	3
Q13: In cases of superobesity or severe obesity-related comorbidities, what is the role of preoperative weight loss, and how can it be safely and effectively achieved?	1	2	2
Q14: For a patient experiencing dumping syndrome after bariatric surgery, outline the nutritional recommendations and dietary modifications that are advised to alleviate symptoms and maintain adequate nutrition.	2	2	3
Q15: How do you mitigate the risk of intraoperative complications, especially in patients with obesity-related anatomical challenges, such as a thickened abdominal wall or enlarged liver?	1	1	3
Q16: What considerations should guide the choice of anesthesia techniques for patients undergoing bariatric surgery, and how do you manage potential anesthesia-related complications?	2	2	3
Q17: What are the key components of a comprehensive perioperative nutritional plan for bariatric surgery patients, and how can we ensure optimal postoperative nutritional support?	2	2	3
Q18: In the case of an adolescent with severe obesity considering bariatric surgery, what are the specific considerations, including surgical technique selection, perioperative planning, and long-term monitoring, to ensure the best outcomes?	1	2	3
Q19: How can healthcare teams effectively identify and manage early postoperative complications, such as leaks, bleeding, or wound infections, to minimize their impact on patient outcomes?	2	2	3
Q20: For a patient with pre-existing GERD who undergoes a sleeve gastrectomy, what measures are taken during the surgery and postoperatively to mitigate the risk of worsening GERD symptoms, and how is the patient monitored for GERD resolution?	2	1	3
Q21: What strategies can be employed to facilitate patient adherence to postoperative dietary guidelines, particularly for those with complex comorbidities or psychosocial challenges?	2	2	3
Q22: How do you manage patients with obesity and a history of eating disorders, and what strategies can be employed to promote a healthy relationship with food post-surgery?	1	2	3
Q23: For patients with superobesity, how can healthcare teams address the challenge of limited mobility and exercise tolerance, both pre- and post-surgery?	2	2	3
Q24: In a case where a patient experiences significant weight regain post-bariatric surgery, what surgical options are available, and how are patients evaluated to determine the most suitable approach for weight loss revision?	2	2	3
Q25: How can healthcare providers work with patients to navigate insurance coverage and address the financial aspects of bariatric surgery, especially when comorbidities are involved?	1	2	3
Q26: In patients with comorbidities, how can revisional surgery be effectively employed to address complications or inadequate weight loss after the initial bariatric procedure?	1	2	3
Q27: For patients with obesity and fertility challenges, how can preoperative counseling and postoperative management be adapted to address their unique needs?	2	2	3
Q28: In a case where a patient’s type 2 diabetes resolves after bariatric surgery, which specific laboratory parameters, such as HbA1c and fasting glucose, are used to monitor their diabetes management, and how are medication adjustments managed in collaboration with endocrinology?	2	2	3
Q29: What long-term health monitoring strategies are most relevant for patients with obesity and comorbidities, and how can early intervention be implemented to optimize patient outcomes?	1	2	3
Q30: In the context of malabsorptive procedures like Roux-en-Y gastric bypass, what should be paid attention to after bariatric surgery?	2	2	3

## Model overview

We assessed three LLMs: ChatGPT-4o (OpenAI), Gemini 2.0 Flash (Google), and DeepSeek-V3 (DeepSeek-AI). Due to the proprietary nature of public technical specifications and their rapid evolution, we described these systems in neutral terms without engaging in speculative parameter discussions.

ChatGPT-4o and Gemini 2.0 Flash are prevalent general-purpose LLMs, with Gemini’s “Flash” variant specifically designed for latency optimization to enhance response speeds [13]. In contrast, DeepSeek-V3 is a newer model that embraces an open-source philosophy, providing publicly accessible checkpoints for select versions. This accessibility facilitates external verification and customization [14].

It’s important to note that none of these models were fine-tuned for MBS by the authors. The comparative performance detailed in this study, conducted on February 3, 2025, should be interpreted with the understanding that model weights and capabilities are subject to change with future updates.

## Model Configuration, Randomization, and Blinding

Each question was entered identically into ChatGPT-4, Gemini 2.0 Flash, and DeepSeek-V3 on 3 February 2025. To achieve consistent and reproducible outputs, we employed deterministic settings (temperature = 0), which effectively minimized stochastic variability. This approach ensured that each model provided a single, repeatable response for each question. In real-world applications, outputs will vary due to differences in prompt phrasing and conversation context, as many systems employ stochastic decoding. This variability is fundamentally different from persistent learning from mistakes, which necessitates explicit retraining, which is not evaluated in this study.

Responses were collected verbatim, and no modifications were made, including truncation; longer responses were retained in full. One author generated and coded the responses, anonymizing them as Model A, B, or C. To prevent position bias, the assignment of models was randomized for every question, such that the order of answers varied across items. Another author, who remained blinded to model identity, created the evaluation survey using Google Forms and disseminated it to participants via email. All expert raters were blinded throughout the study, and the survey platform presented responses in randomized order to minimize recognition effects. The randomization key was stored securely and disclosed only after completion of the statistical analysis.

## Participants and Recruitment

Eligible participants were MBS surgeons with at least ten years of clinical experience in the field. Invitations were emailed to MBS experts worldwide beginning in March 2025, with reminder emails sent in April, May, and June 2025. Survey administration emphasized blinding, randomization, and ethical safeguards. Experts provided informed consent, rated confidential and anonymized responses presented in randomized order, and were offered optional collaborative authorship upon completion. The survey instructions, rating items, and response options was administered in English.

The study was closed on 17 July 2025. Each invitation included a link to the evaluation survey, which was hosted on Google Forms. To encourage transparency and collaboration, all invited experts were also offered the option of collaborative authorship upon completion of the survey and provision of informed consent. Experts who accepted were listed as collaborative authors. Authors who managed coding, survey preparation, or held the randomization key were excluded from participation to maintain blinding and avoid conflicts.

### Outcome Measures

The primary outcome was the mean expert rating of each model’s responses using the rubric of Leng et al. [11]. Scores ranged from 0 to 3, where 0 = entirely inaccurate/irrelevant, 1 = partially correct but substantially incomplete, 2 = largely correct and clinically acceptable, and 3 = fully guideline concordant.

For scope clarity, this outcome operationalized the QUEST dimension Quality of Information [15]; instruments for Expression Style and Persona, Safety and Harm, and Trust and Confidence were not administered and lay outside the study scope.

## Sample Size Calculation

A priori sample size estimation was conducted using G*Power version 3.1 to determine the number of expert raters required for the repeated-measures ANOVA design with three within-subject conditions (ChatGPT-4o, Gemini 2.0 Flash, and DeepSeek-V3). Parameters were set at a two-tailed α of 0.05, power (1–β) of 0.95, and a medium effect size (f = 0.25). The analysis indicated that 43 expert raters would be required. To account for potential nonresponse or incomplete participation, the target was increased by 20%, yielding a final minimum sample size of 52 expert raters.

### Statistical Analysis

Data distribution was examined using the Shapiro–Wilk test. A one-way repeated-measures ANOVA was conducted to test for differences in mean ratings between ChatGPT-4o, Gemini 2.0 Flash, and DeepSeek-V3. Bonferroni-adjusted pairwise comparisons identified specific differences between models. Sphericity was tested using Mauchly’s test. When the assumption of sphericity was violated, Greenhouse–Geisser corrected degrees of freedom were reported. Inter-rater reliability was assessed in two ways: (1) Internal consistency of expert raters for each model using Cronbach’s alpha. (2) Absolute agreement between expert raters using the two-way mixed effects intraclass correlation coefficient (ICC). All analyses were performed in IBM SPSS Statistics v25. In addition, we conducted an exploratory, descriptive mapping of the QUEST framework to clarify which dimensions were directly addressed by the design [15]; this mapping was not pre-specified and involved no additional hypothesis testing.

### Authorship Criteria and Ethical Considerations

The principal authors of this study were directly involved in the conception and design of the research, adaptation of the question framework, coding and anonymization of responses, survey preparation and distribution, statistical analysis, interpretation of findings, and manuscript preparation and revision. Expert raters, who contributed solely by completing the survey and rating the responses, were granted the option of collaborative authorship upon their consent. This distinction was made to ensure that all full authors met the criteria for substantial contributions to the study’s design, analysis, and writing, while also acknowledging the critical input of survey respondents through collaborative authorship.

Before participation, expert raters provided informed consent, with the survey presenting anonymized responses designated as Model A/B/C in a randomized sequence. Individual ratings remained anonymous to the investigators, and the raw identity-rating linkages were never accessed. Consent and completion of the survey permitted listings as collaborative authors; importantly, authorship was not dependent on the content or scores of the ratings provided. To maintain blinding, investigators involved in coding, survey setup, or the generation of the randomization key were excluded from the rating process.

## Results

### Response Rate

**Fig. 1:**
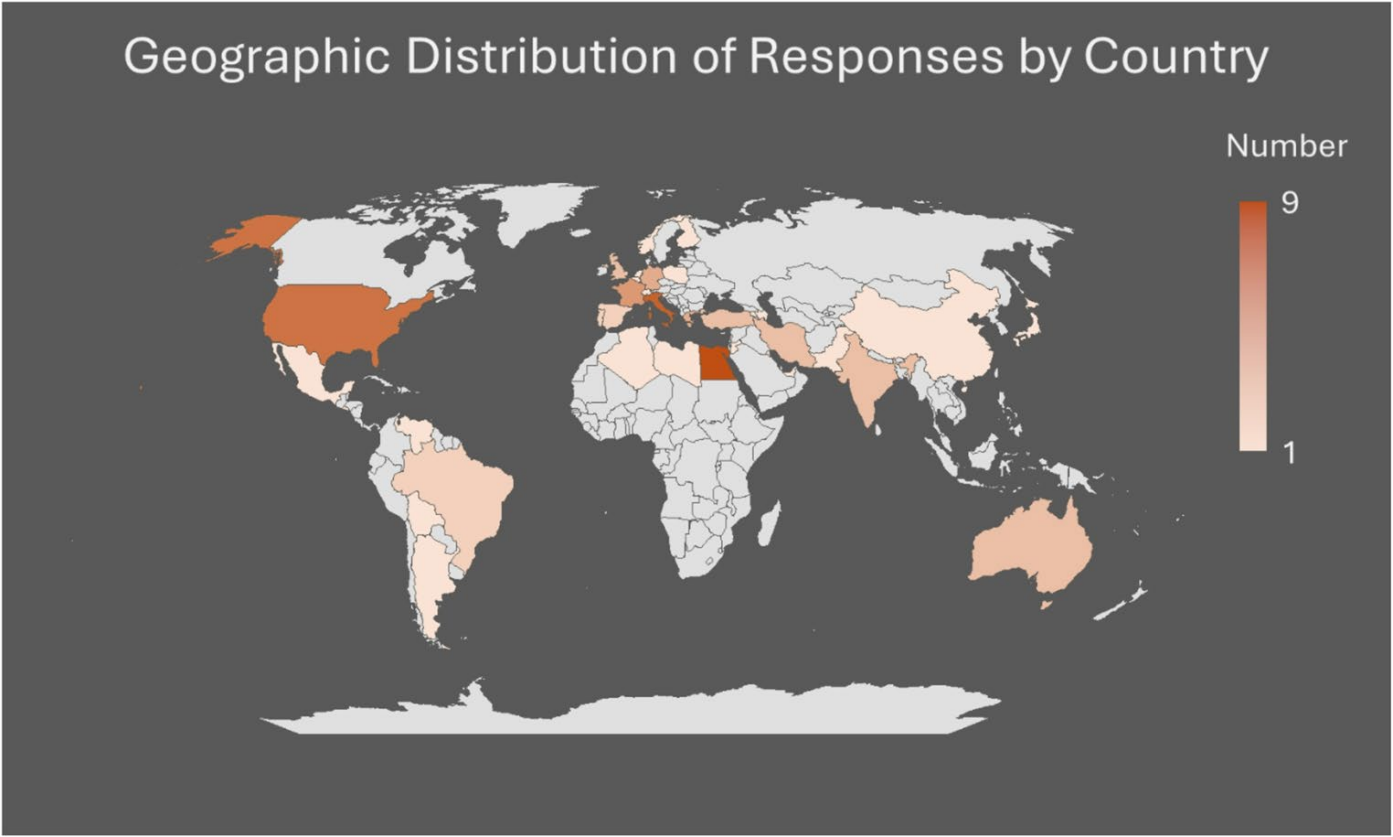
Geographic Distribution of survey responses across countries. Egypt contributed the highest number of responses (n = 9), followed by Italy (n = 8), the USA (n = 7), and France (n = 5)

A total of 93 MBS experts from 49 different countries completed the evaluation survey, exceeding the minimum sample size requirement (Fig. 1). All analysis was conducted on this final cohort. Figure 1 presents an aggregated, non-identifying summary of respondent geographic distribution.

### Descriptive Statistics

**Fig. 2:**
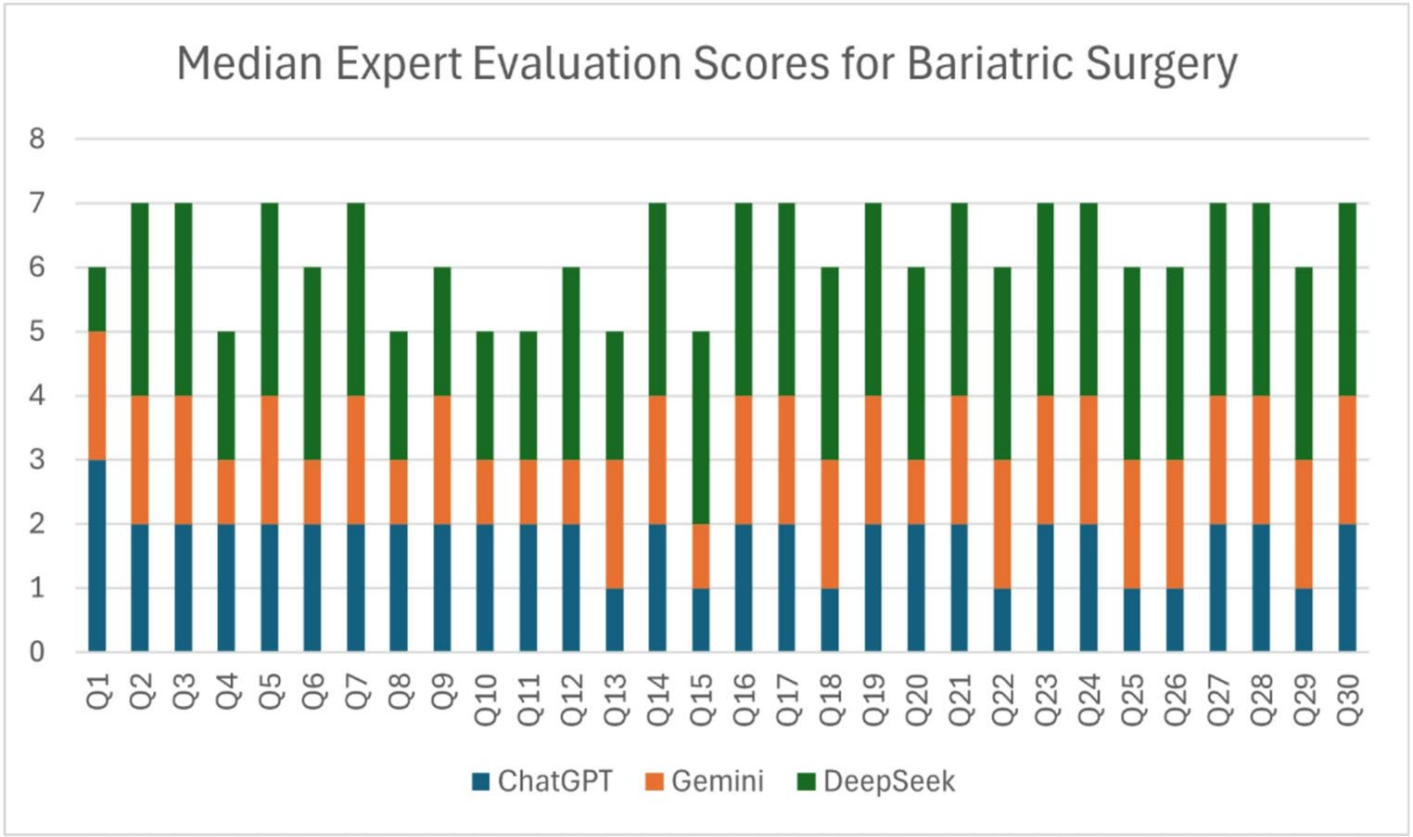
Median expert evaluation scores for bariatric surgery responses generated by large language models. Median scores (range 0–3) across 30 evaluation items (Q1–Q30) comparing ChatGPT (blue), Gemini (orange), and DeepSeek (green). DeepSeek consistently achieved higher expert evaluation scores relative to Gemini and ChatGPT across most questions

Table 1 presents the median scores assigned to responses generated by ChatGPT-4o, Gemini 2.0 Flash, and DeepSeek-V3 for 30 bariatric surgery–related clinical questions. For ChatGPT-4o, 25 (83.3%) responses received a score of 2, and 5 (16.7%) responses received a score of 1. For Gemini 2.0 Flash, 1 (3.3%) response received a score of 3, 16 (53.3%) responses received a score of 2, and 13 (43.3%) responses received a score of 1 (Table 1). For DeepSeek-V3, 24 (80.0%) responses received a score of 3, 6 (20.0%) responses received a score of 2 (Fig. 2). Mean (± SD) ratings were highest for DeepSeek-V3 (2.44 ± 0.40), followed by ChatGPT-4o (1.79 ± 0.46), and Gemini 2.0 Flash (1.63 ± 0.47).

### Reliability Analysis

The reliability analysis demonstrated excellent internal consistency for all three models, with Cronbach’s alpha values exceeding 0.90 (ChatGPT-4o α = 0.953, Gemini 2.0 Flash α = 0.928, DeepSeek-V3 α = 0.960), indicating that expert raters showed highly consistent scoring patterns within each model. The intraclass correlation coefficients (ICC) values for average measures were high (ChatGPT-4o = 0.929, Gemini 2.0 Flash = 0.889, DeepSeek-V3 = 0.942), indicating good to excellent agreement when ratings were aggregated across the full panel of 93 expert raters.

Internal consistency was excellent for all three models (Cronbach’s α > 0.90: ChatGPT 4o = 0.953; Gemini 2.0 Flash = 0.928; DeepSeek-V3 = 0.960), indicating that expert raters showed highly consistent scoring patterns within each model. The intraclass correlation coefficients (ICC) values for average measures were high (ChatGPT-4o = 0.929, Gemini 2.0 Flash = 0.889, DeepSeek-V3 = 0.942), indicating good to excellent agreement when ratings were aggregated across the full panel of 93 expert raters.

In a descriptive, non-pre-specified, mapping to the QUEST framework [15], our design directly addressed Quality of Information via expert accuracy ratings and only indirectly touched on Understanding; Expression, Safety, and Trust were not measured.

### Effect of Model Type on Ratings

Mauchly’s test indicated that the assumption of sphericity was violated, χ²(2) = 37.72, *p* < 0.001. Therefore, Greenhouse–Geisser correction (ε = 0.747) was applied. A repeated-measures ANOVA indicated a significant main effect of model on ratings, F(1.493, 137.38) = 179.58, *p* < 0.001, partial η² = 0.661. Bonferroni-adjusted pairwise comparisons revealed that ChatGPT-4o was rated significantly higher than Gemini 2.0 Flash (Mean difference (MD) = 0.156, 95% CI (0.077, 0.234), *p* < 0.001, Cohen’s *d* = 0.33) (Table 2). DeepSeek received significantly higher ratings than both ChatGPT-4o (MD = 0.654, 95% CI (0.544, 0.764), *p* < 0.001, Cohen’s *d* = 1.51) and Gemini (MD = 0.810, 95% CI (0.674, 0.946), *p* < 0.001, Cohen’s *d* = 1.84) (Table 2). These findings indicate a clear ranking in mean ratings, with DeepSeek rated highest, followed by ChatGPT-4o, and Gemini 2.0 Flash receiving the lowest ratings.

**Table 2:** Pairwise Comparisons of Model Ratings with Bonferroni Adjustment

Comparison	Mean Diff	95% CI (Lower–Upper)	Cohen’s d	*p*-value
ChatGPT-4o vs. Gemini 2.0 Flash	0.156	0.077–0.234	0.33	**< 0.001**
DeepSeek-V3 vs. ChatGPT-4o	0.654	0.544–0.764	1.51	**< 0.001**
DeepSeek-V3 vs. Gemini 2.0 Flash	0.810	0.674–0.946	1.84	**< 0.001**

## Discussion

Our blinded evaluation identified statistically and clinically meaningful performance differences among ChatGPT-4o, Gemini 2.0 Flash, and DeepSeek-V3 on MBS questions. DeepSeek-V3 achieved the highest scores for guideline-concordance, followed by ChatGPT-4o, then Gemini 2.0 Flash. In our 30-item MBS benchmark, DeepSeek-V3 received the highest proportion of ratings of ‘3’ (fully guideline-concordant per our rubric), suggesting closer alignment with contemporary MBS guidance for these prompts and a potential practical advantage in this domain. These findings should be interpreted as domain-specific and time-bound to the model snapshots used during response generation (3 February 2025) and to the expert-rating period (March–July 2025). Rather than implying universal superiority of any system, the results highlight that newer or less widely adopted LLMs can, in certain specialized domains, outperform established counterparts. This underscores the need for domain-specific validation before LLMs are integrated into clinical decision-making in MBS.

The superior performance of DeepSeek-V3 within this specialized domain challenges the assumption that mainstream models are always more reliable. By introducing DeepSeek-V3 alongside ChatGPT-4o and Gemini 2.0 Flash, our study broadens evaluation beyond ChatGPT-centric literature [6, 8, 9, 11, 12] and reinforces that not all LLMs are equally suited to high-stakes applications, particularly where precision, adherence to guidelines, and up-to-date knowledge are essential [16, 17].

Evidence outside MBS supports this domain-specific observation. A recent Nature Medicine benchmark compared DeepSeek-V3 and DeepSeek-R1 against ChatGPT-4o and Gemini 2.0 across diverse diagnostic and treatment scenarios, finding that DeepSeek models performed comparably or better on expert-rated accuracy [18]. While such findings validate the general clinical competence of DeepSeek-V3, they do not substitute for MBS-specific evaluation, underscoring the importance of domain-focused benchmarks like our evaluation.

One plausible explanation for these performance differences lies in the models’ development philosophies. ChatGPT-4o and Gemini follow closed-source, commercially maintained pathways that prioritize broad linguistic fluency, alignment with human feedback, and user-friendly conversational design [14]. In contrast, DeepSeek-V3 emphasizes reinforcement learning, cost efficiency, structured reasoning, and transparency, though sometimes at the expense of conversational polish and multilingual breadth [14]. These differences may partly account for the stronger performance of DeepSeek-V3 in our evaluation, although further work is required to establish causality. As we did not inspect proprietary training corpora, potential explanations remain speculative; factors such as the recency and breadth of biomedical data, differences in safety alignment, and the latency-optimized design of Gemini 2.0 Flash may also have contributed. Taken together, these factors motivate a domain-specific view of utility that extends beyond accuracy alone [19].

The integration of LLMs into healthcare presents both immense opportunities and significant challenges. In MBS specifically, prior work shows that ChatGPT-4o can simplify patient-education materials to recommended readability levels while preserving accuracy, although simplification reduced comprehensiveness in some models [19]. LLMs have the potential to revolutionize medical education, assist in clinical decision support, streamline administrative tasks, and even enhance patient communication [20, 21]. For instance, their ability to rapidly process vast amounts of medical literature can aid clinicians in being updated with the latest research and guidelines, thereby improving diagnostic accuracy and treatment efficacy. However, the deployment of these powerful tools is not without its caveats. Concerns regarding data privacy, algorithmic bias, and the potential for misinformation remain critical considerations [22, 23]. Our study, by demonstrating varying levels of accuracy among different LLMs, reinforces the need for continuous evaluation and validation to ensure that these technologies are not only effective but also safe and ethical in their application within the complex medical landscape.

The findings presented have direct implications for MBS-related clinical decision-making, particularly as LLMs become more embedded in medical workflows. Prospective randomized evidence in bariatric patient communications found that 64.9% of patients preferred ChatGPT-4o responses over physician replies for clarity, completeness, and empathy, with clinicians subsequently correcting outputs when needed—supporting a supervised “LLM-draft + expert-review” workflow rather than unsupervised use [24]. The observed variability in performance among ChatGPT-4o, Gemini 2.0 Flash, and DeepSeek-V3 highlights the critical need for rigorous, domain-specific validation in MBS before these models are considered for use in real-world clinical environments. While LLMs provide considerable advantages in terms of rapid knowledge retrieval, decision support, and patient education, their shortcomings, especially in contexts demanding strict compliance with evidence-based guidelines and complex clinical reasoning, must be fully recognized and rectified [25, 26]. In practice, this argues for supervised workflows (LLM-draft + expert-review) rather than unsupervised deployment, particularly for high-stakes decisions [19, 24].

The widespread adoption of LLMs in medicine faces several challenges. These include concerns about data privacy and security, the potential for algorithmic bias leading to health disparities, and the ‘black box’ nature of some models, which can hinder interpretability and trust [27, 28]. Furthermore, the dynamic nature of medical knowledge necessitates continuous updating and retraining of LLMs to maintain their accuracy and relevance. The ethical implications of delegating critical medical tasks to AI, such as diagnosis or treatment planning, also require careful consideration and robust regulatory frameworks. Our study, although limited to accuracy in MBS, contributes to this discussion by highlighting performance variability, which could have significant consequences if less accurate models were deployed without adequate safeguards.

### Strengths

This study exhibits several notable strengths that bolster the internal validity and reliability of its findings. Firstly, the implementation of a randomized, blinded, repeated-measures design effectively mitigates bias and facilitates direct head-to-head comparisons of LLM performance, addressing a methodological rigor often absent in preliminary evaluations of AI applications in medicine. The integration of a validated question set, adapted from the work of Leng et al. [11], reinforces the study’s internal validity by ensuring that the prompts hold clinical relevance and consistency.

Secondly, featuring DeepSeek-V3, an under-explored yet highly capable LLM, alongside established models like ChatGPT-4o and Gemini 2.0 Flash, provides insights into the performance landscape of emerging AI technologies within the context of MBS. This comparative methodology yields an informative snapshot of the current capabilities of LLMs in this field, serving as a foundation for subsequent research and development initiatives.

Moreover, the study’s recruitment of a sizable panel of 93 MBS experts for evaluation, exceeding the pre-determined sample size, ensures robust statistical power and enhances the reliability of expert ratings. The study demonstrates internal consistency and inter-rater reliability, thereby affirming the integrity of the scoring process.

Finally, by concentrating on a specialized medical domain like MBS, the research provides a concrete case for evaluating LLM performance in high-stakes clinical contexts. This specificity not only underscores the findings’ relevance to the implementation of AI in MBS surgical practice but also hints at the broader necessity for rigorous validation and investigation into performance variability across diverse medical fields, necessitating further empirical assessment in future studies.

### Limitations

Despite its significant findings, this study has several limitations relevant to the potential application of LLMs in MBS and, cautiously, in other domains. First, the evaluation was based on 30 curated clinical questions, which, although validated, cannot capture the full breadth and complexity of real-world MBS scenarios. Broader case-mix and multi-faceted clinical vignettes are needed to reflect actual practice more accurately.

Second, although deterministic settings (temperature = 0) were applied to produce reproducible outputs, this design meant that only one response per question per model was evaluated. While this controlled sampling-related randomness in our study, real-world users may encounter variability across repeated interactions due to differences in phrasing or prior conversational context, and because many platforms operate with non-deterministic decoding. This run-to-run variability should not be conflated with persistent “learning from mistakes”: in typical deployment, apparent improvement within a session mainly reflects in-context conditioning rather than model weight updates, and our study did not test post-feedback adaptation or iterative correction. Future studies should therefore incorporate multi-sample responses to better capture stability and reproducibility, as emphasized by Lee et al. [29].

Third, our scoring rubric emphasized guideline-concordance but did not formally classify error types. Following recommendations from emerging evaluation frameworks, categorizing errors into logical, informational, or statistical domains could provide deeper insight into the limitations and failure patterns of LLMs [29].

Moreover, the ratings reflect expert MBS surgeons’ judgments rather than empirical patient outcomes, limiting their evidence and generalizability. While valuable for face validity, these evaluations rank low on the hierarchy of evidence.

The responses provided by LLMs are not always consistent and can change over time. This variability means that the same posed question to the same model at different times may yield different answers, undermining the reliability required for clinical decision-making. Accordingly, our findings should be interpreted as a time-stamped benchmark under controlled conditions rather than a guarantee of future performance; longitudinal testing is required to assess model drift as platforms undergo updates.

Ultimately, the study primarily focused on accuracy and adherence to guidelines. Other important aspects, such as handling ambiguity, adapting to rapidly evolving evidence, and safeguarding against bias, were not measured. Longitudinal assessments are warranted to evaluate performance drift as models undergo continuous updates. Ethical considerations, including patient safety, equity, and transparency, must also remain central to the responsible deployment of LLMs in surgery. Despite all these limitations, this is the first study attempting to study these platforms in a randomized and blinded manner.

### Alignment with Evaluation Frameworks

The study aligns closely with emerging evaluation frameworks for healthcare LLMs [15]. The proposed QUEST framework encompasses five dimensions: Quality of Information, Understanding and Reasoning, Expression Style and Persona, Safety and Harm, and Trust and Confidence [15]. Our design explicitly addressed Quality of Information through expert accuracy ratings and partially captured Understanding and Reasoning by rewarding guideline-concordant logic. However, we did not assess higher-order reasoning processes or model explainability beyond guideline concordance.

Expression Style and Persona were not evaluated, leaving aspects such as readability, clarity, and communication style unexplored. Safety and Harm were only indirectly considered via correctness, without analysis of potentially biased, incomplete, or misleading outputs. Trust and Confidence were inferred from expert scorings but not systematically measured using structured trust or reliability scales [29]. Future studies should expand to explicitly capture all QUEST dimensions, ensuring a comprehensive, standardized evaluation of LLMs in surgical contexts [15, 29].

### Future Directions

Future research should prioritize evaluating emerging LLMs and AI agent systems across the spectrum of MBS applications, with emphasis on accurate, consistent, and clinically pertinent outputs. This should extend beyond single-snapshot testing to include multi-sample stability metrics, vignette-based assessments of complex comorbidity and trade-offs (including conflicting guideline scenarios), and longitudinal reassessment to quantify performance drift as platforms update. In parallel, examining architectures and training methodologies associated with stronger task performance (as observed for DeepSeek-V3) may guide safer domain adaptation [30].

To advance this agenda, we recommend an MBS-specific Retrieval-Augmented Generation (RAG) system anchored to vetted guidelines and peer-reviewed evidence with explicit citation of retrieved sources, developed through collaboration with LLM developers, clinicians, and IFSO, supported by governance for source curation, update cadence, audit trails, and safety monitoring. Feasibility, including maintenance burden, licensing/access to full-text resources, compute/hosting requirements, and operational cost, should be evaluated explicitly to ensure clinical trustworthiness and practical sustainability.

## Conclusion

This randomized, blinded evaluation demonstrated that large language models differ significantly in their ability to generate guideline-concordant responses within the domain of metabolic and bariatric surgery. DeepSeek-V3 outperformed both ChatGPT-4 and Gemini 2.0 Flash, underscoring that emerging models can, in certain specialized contexts, surpass more established systems. These findings are domain-specific and reflect the state of the models at the time of querying. They reinforce the need for ongoing, domain-focused validation before LLMs are integrated into clinical decision-making in bariatric surgery. 

## Supplementary Information

Below is the link to the electronic supplementary material.

Supplementary Material 1 (DOCX 14.8 KB)

## Data Availability

De-identified item-level ratings, the question set, scoring rubric, randomization procedure, and analysis outputs are available from the corresponding author upon reasonable request.

## Code Availability

All analysis scripts (including the repeated-measures ANOVA workflow, effect-size calculations, robustness checks, and figure-generation code) are available from the corresponding author upon reasonable request. No proprietary software was required.

## Declarations

### Use of artificial intelligence

No generative AI systems were used to generate, analyze, or interpret the study data or to write the scientific content of this manuscript.
