# Supplementary material for: Accuracy and Knowledge Base Evaluation of ChatGPT-4o, Gemini-2.0-Flash, and DeepSeek-V3 in Metabolic and Bariatric Surgery: an Expert-Rated Blinded Study
Source: Obes Surg. 2026 Mar 25;36(5):2160–71. doi: 10.1007/s11695-026-08562-z (PMC13222281; doi:10.1007/s11695-026-08562-z)
Supplement: Supplementary file 1 — Supplementary Material 1 (DOCX 14.8 KB) [file 11695_2026_8562_MOESM1_ESM.docx]

# Electronic Supplementary Material 2 – Example of LLM responses Prompts

This supplement provides one representative example from the evaluated question set (Q1), showing the prompt and the full verbatim outputs produced by the three assessed LLMs. Responses are reproduced as originally generated (no editing for content). They were generated on 3 February 2025 and later presented to expert raters for blinded scoring. Full response gradings are provided in Table 1 in the manuscript.

## Prompt (Q1)

What is the ideal BMI range for patients to be considered suitable candidates for bariatric surgery, and how does this range change with different surgical procedures?

## ChatGPT-4o output

The ideal BMI range for bariatric surgery is typically ≥40, or ≥35 with obesity-related comorbidities. Recent guidelines expand eligibility to ≥35 without comorbidities and ≥30 with significant comorbidities (e.g., type 2 diabetes), with criteria generally consistent across procedures, though specific recommendations may vary based on surgical type and patient health.

## Gemini 2.0 Flash output

The ideal BMI for bariatric surgery is generally 40 or higher, or 35 or higher with serious weight-related health problems. Some patients with a BMI between 30 and 34.9 may qualify if they have serious health problems.

## DeepSeek-V3 output

The ideal BMI range for bariatric surgery candidates is ≥40 kg/m² or ≥35 kg/m² with obesity-related comorbidities. For certain procedures like gastric banding, a lower BMI (30–35 kg/m²) may be considered in select cases, while malabsorptive procedures like duodenal switch are often recommended for patients with a BMI ≥50 kg/m² due to greater weight loss needs.
